# Supplementary material for: The effects of genital myiasis on the diversity of the vaginal microbiota in female Bactrian camels
Source: BMC Vet Res. 2022 Mar 5;18:87. doi: 10.1186/s12917-022-03189-5 (PMC8897907; doi:10.1186/s12917-022-03189-5)
Supplement: Supplementary file 5 — Additional file 5. [file 12917_2022_3189_MOESM5_ESM.zip › MPL201709200_16s_yy/Treat1/css/js_css/jsPDF-master.zip!/jsPDF-master/old-tests/015_splittext.pdf]

Lorem ipsum dolor sit amet, consectetur adipiscing elit. Phasellus id eros turpis. Vivamus tempor urna vitae sapien mollis molestie. Vestibulum in lectus non enim bibendum laoreet at at libero. Etiam malesuada erat sed sem blandit in varius orci porttitor. Sed at sapien urna. Fusce augue ipsum, molestie et adipiscing at, varius quis enim. Morbi sed magna est, vel vestibulum urna. Sed tempor ipsum vel mi pretium at elementum urna tempor. Nulla faucibus consectetur felis, elementum venenatis mi mollis gravida. Aliquam mi ante, accumsan eu tempus vitae, viverra quis justo.

Proin feugiat augue in augue rhoncus eu cursus tellus laoreet. Pellentesque eu sapien at diam porttitor venenatis nec vitae velit. Donec ultrices volutpat lectus eget vehicula. Nam eu erat mi, in pulvinar eros. Mauris viverra porta orci, et vehicula lectus sagittis id. Nullam at magna vitae nunc fringilla posuere. Duis volutpat malesuada ornare. Nulla in eros metus. Vivamus a posuere libero. Proin ut placerat lectus. Duis vitae felis eget eros feugiat congue. Donec tempus, arcu a consectetur mollis, magna augue consequat augue, vel blandit eros ligula non tellus.

Lorem ipsum dolor sit amet, consectetur adipiscing elit. Phasellus id eros turpis. Vivamus tempor urna vitae sapien mollis molestie. Vestibulum in lectus non enim bibendum laoreet at at libero. Etiam malesuada erat sed sem blandit in varius orci porttitor. Sed at sapien urna. Fusce augue ipsum, molestie et adipiscing at, varius quis enim. Morbi sed magna est, vel vestibulum urna. Sed tempor ipsum vel mi pretium at elementum urna tempor. Nulla faucibus consectetur felis, elementum venenatis mi mollis gravida. Aliquam mi ante, accumsan eu tempus vitae, viverra quis justo.

Proin feugiat augue in augue rhoncus eu cursus tellus laoreet. Pellentesque eu sapien at diam porttitor venenatis nec vitae velit. Donec ultrices volutpat lectus eget vehicula. Nam eu erat mi, in pulvinar eros. Mauris viverra porta orci, et vehicula lectus sagittis id. Nullam at magna vitae nunc fringilla posuere. Duis volutpat malesuada ornare. Nulla in eros metus. Vivamus a posuere libero. Proin ut placerat lectus. Duis vitae felis eget eros feugiat congue. Donec tempus, arcu a consectetur mollis, magna augue consequat augue, vel blandit eros ligula non tellus.

Lorem ipsum dolor sit amet, consectetur adipiscing elit. Phasellus id eros turpis. Vivamus tempor urna vitae sapien mollis molestie. Vestibulum in lectus non enim bibendum laoreet at at libero. Etiam malesuada erat sed sem blandit in varius orci porttitor. Sed at sapien urna. Fusce augue ipsum, molestie et adipiscing at, varius quis enim. Morbi sed magna est, vel vestibulum urna. Sed tempor ipsum vel mi pretium at elementum urna tempor. Nulla faucibus consectetur felis, elementum venenatis mi mollis gravida. Aliquam mi ante, accumsan eu tempus vitae, viverra quis justo.

Proin feugiat augue in augue rhoncus eu cursus tellus laoreet. Pellentesque eu sapien at diam porttitor venenatis nec vitae velit. Donec ultrices volutpat lectus eget vehicula. Nam eu erat mi, in pulvinar eros. Mauris viverra porta orci, et vehicula lectus sagittis id. Nullam at magna vitae nunc fringilla posuere. Duis volutpat malesuada ornare. Nulla in eros metus. Vivamus a posuere libero. Proin ut placerat lectus. Duis vitae felis eget eros feugiat congue. Donec tempus, arcu a consectetur mollis, magna augue consequat augue, vel blandit eros ligula non tellus.
